# Radiation-induced chondrosarcoma of the scapula after radiotherapy for lung cancer: a case report and review of the literature

**DOI:** 10.1186/s13256-018-1606-2

**Published:** 2018-03-05

**Authors:** Abdelfettah Zidane, Adil Arsalane, Mohammed Lahkim, Issam Lalya, Abderrahim Ktaibi, Ismail Essadi

**Affiliations:** 1 Thoracic surgery, Ibn Sina Military Hospital, Faculty of Medicine and Pharmacy Mohamed VI, Caddy Ayyad University, Marrakesh, Morocco; 2 General Surgery, Ibn Sina Military Hospital, Faculty of Medicine and Pharmacy Mohamed VI, Caddy Ayyad University, Marrakesh, Morocco; 3 Radiation Oncology, Mohammed V Military Hospital, Faculty of Medicine and Pharmacy Mohamed VI, Caddy Ayyad University, Marrakesh, Morocco; 4 Anatomopathology, Ibn Sina Military Hospital, Faculty of Medicine and Pharmacy Mohamed VI, Caddy Ayyad University, Marrakesh, Morocco; 5 Medical Oncology, Ibn Sina Military Hospital, Faculty of Medicine and Pharmacy Mohamed VI, Caddy Ayyad University, Marrakesh, Morocco

**Keywords:** Radiotherapy, Sarcomas, Scapula, Chondrosarcoma

## Abstract

**Background:**

Radiotherapy associated with chemotherapy is a well-established treatment modality for locally advanced non-small cell lung cancers. Radiation-induced second malignancies, particularly radiation-induced sarcomas, are rare. Some authors reported a recent increase in the incidence of this rare complication, especially because of the improved prognosis and survival of patients after radiotherapy. Pathogenic mechanisms of radiation-induced sarcomas are poorly understood. However, diagnosis criteria are well established. Treatment options must be discussed and adapted to the patient’s profile. Surgery in irradiated tissue is challenging, with limited treatment options with chemotherapy and radiotherapy.

**Case presentation:**

We report the case of a 62-year-old Moroccan man diagnosed as having chondrosarcoma of his right scapula, who was irradiated 10 years ago for stage IIIB non-small cell lung cancer. This case was managed by a complete resection of the tumor with good functional and oncological outcomes. To the best of our knowledge, the scapular location of radiation-induced sarcoma after irradiation for lung cancer has never been described in the literature.

**Conclusion:**

Radiation-induced sarcoma of the scapula represents a rare situation that must be actively researched to have access to an optimal therapeutic approach.

## Background

Radiation therapy (RT) plays a significant role in the management of thoracic tumors [1]. Most of these tumors require RT as a local measure for definitive treatment of medically inoperable or surgically unresectable disease, or as part of a multimodality regimen for locally advanced disease [1, 2]. The standard care for stage III unresectable disease is combined chemoradiotherapy [1, 2].

The occurrence of radiation-induced sarcoma (RIS) in the scapula after RT for lung cancer is a very rare complication [3]. With improved oncologic outcomes, post-irradiation sarcomas are increasingly seen in long-term survivors with an estimated risk of up to 0.3% [4]. The spectrum of radiation-induced tumors includes soft tissue sarcoma, osteosarcoma, squamous cell carcinoma (SCC), leukemia, and neuroendocrine carcinomas, which may develop in the head and neck, esophagus, lung, or stomach [5]. We report here a case of chondrosarcoma of the right scapula occurring 10 years after completion of chemoradiotherapy for stage IIIB SCC developed in the upper lobe of the right lung.

## Case presentation

**Fig. 1:**
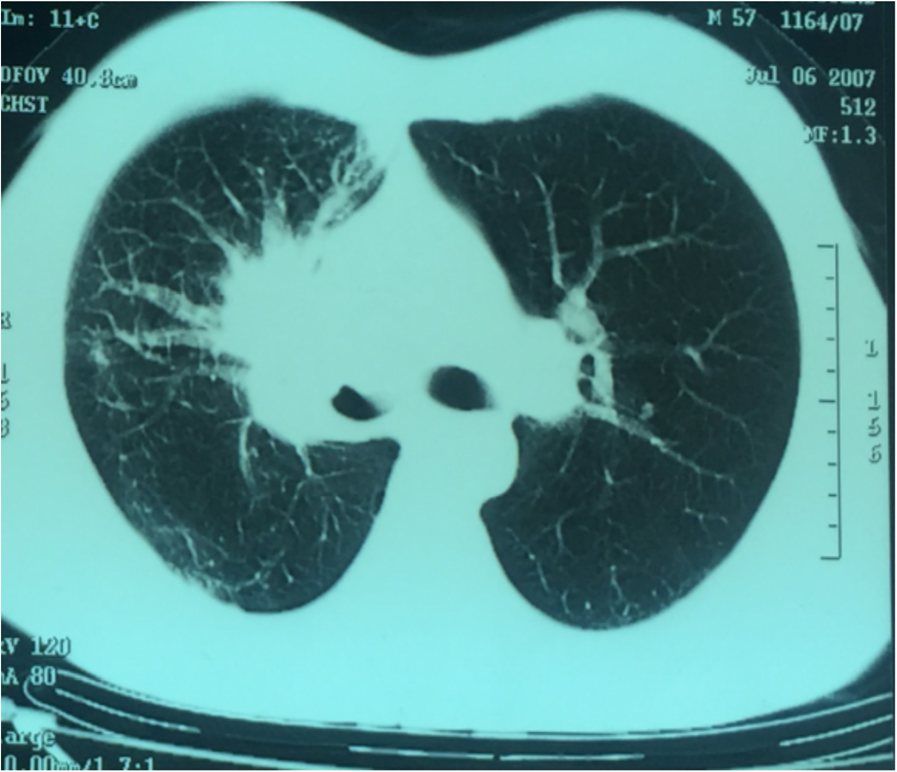
Scannographic cut showing a mediastinopulmonary process stage IIIB

**Fig. 2:**
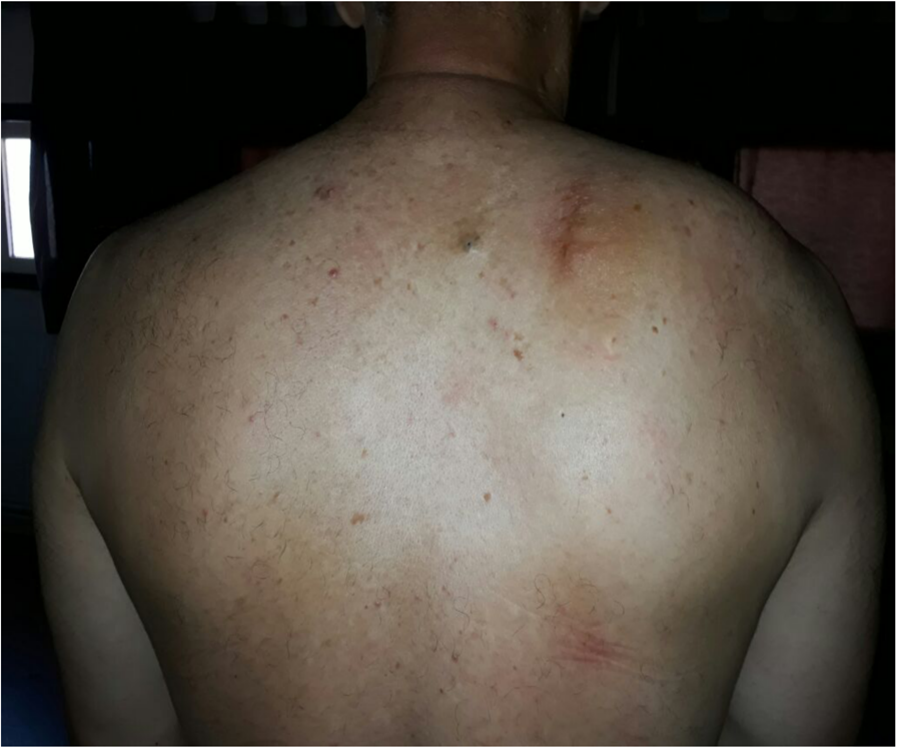
Right scapular mass

**Fig. 3:**
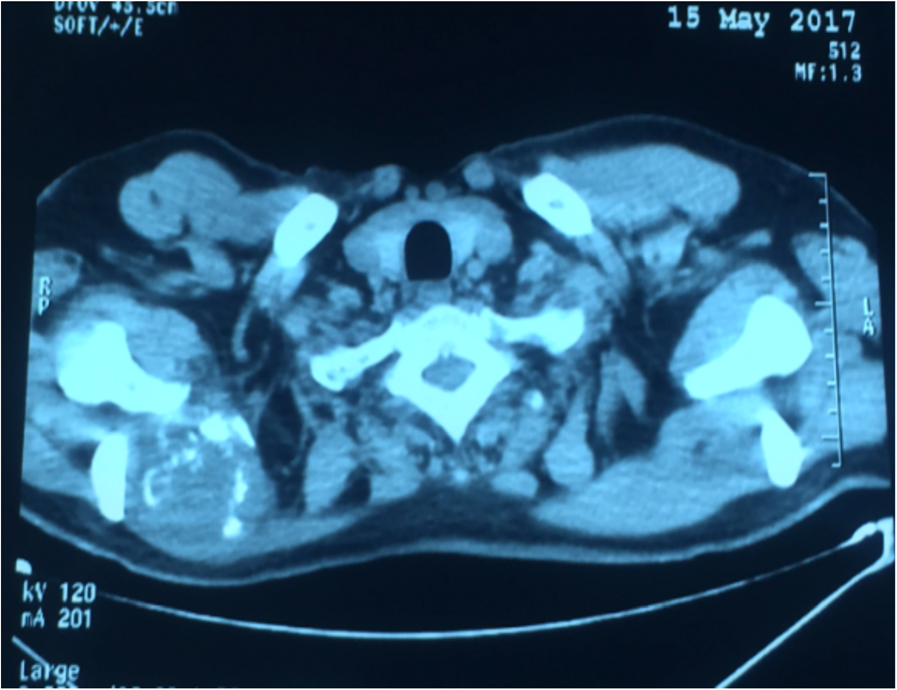
Chest computed tomography scan showing a tumor process on the right scapula

**Fig. 4:**
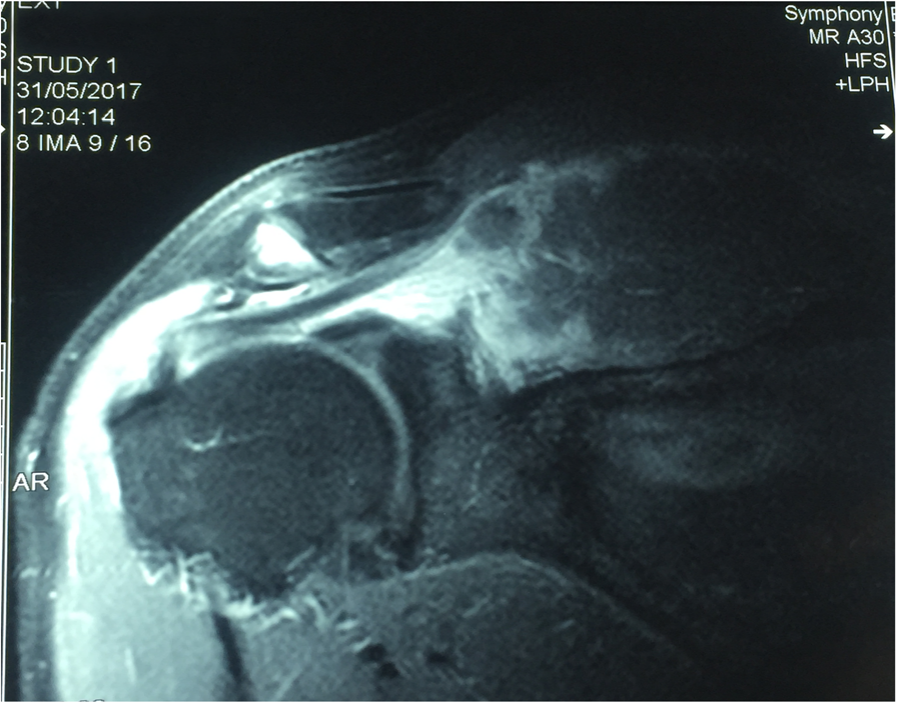
Magnetic resonance imaging of the scapular region with malignant tumor process centered on the supraspinous and infraspinous muscles

**Fig. 5:**
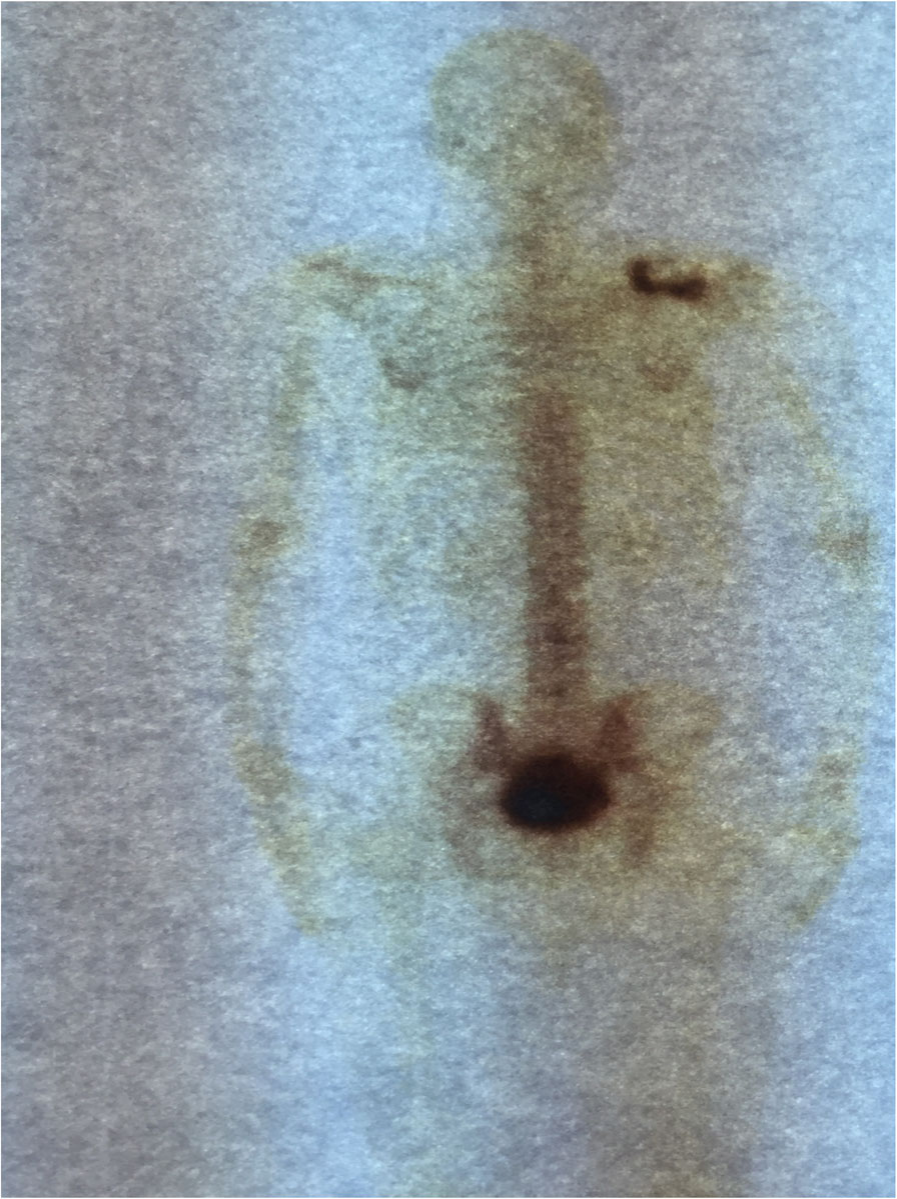
Bone scintigraphy with hyperfixation focus at the right scapula

**Fig. 6:**
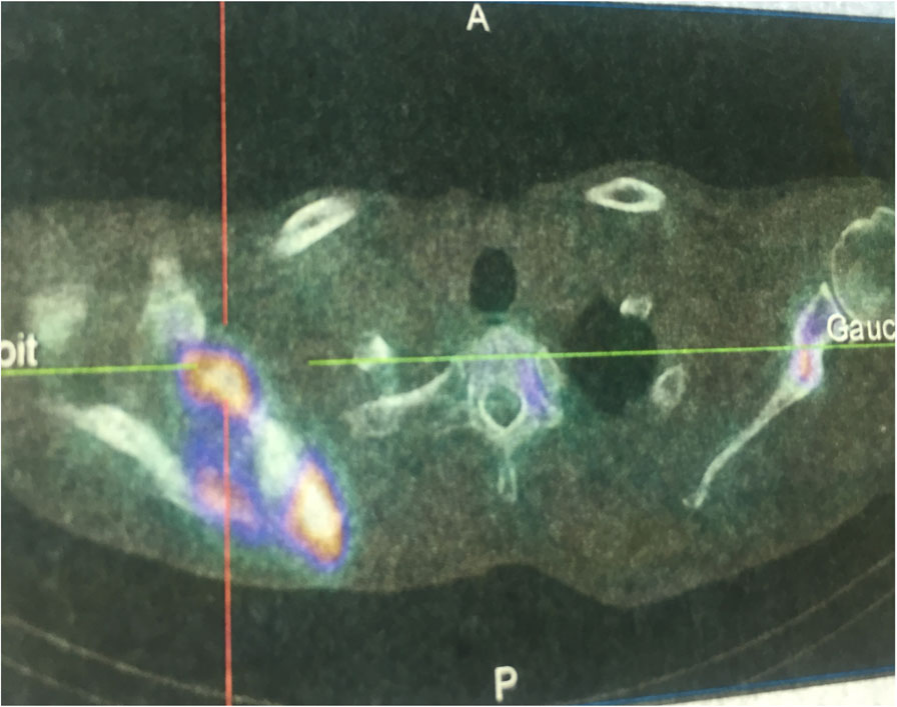
Single-photon emission computed tomography-computed tomography with osteocondensing and lytic aspect in the spine of the right scapula

**Fig. 7:**
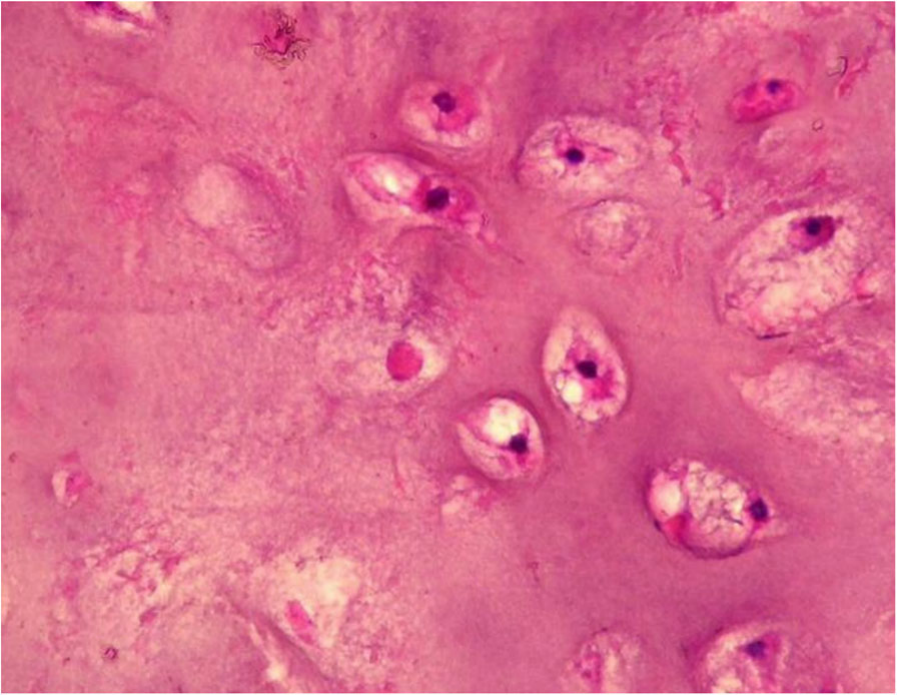
Histopathological examination of the biopsy in favor of grade 1 chondrosarcoma

A 62-year-old Moroccan man, retired from the army, married, father of five children, a chronic tobacco smoker who did not consume alcohol, without any medical history, underwent concomitant chemoradiation for stage IIIB SCC of his right lung in 2007 (Fig. 1). The protocol included radiotherapy using gamma photons of 1.25 Mv energy, delivered at a total dose of 60 Gy, 2 Gy by fraction, in 6 weeks, with two opposed fields including the right scapula. In combination with the RT we used chemotherapy according to the vinorelbine-cisplatin regimen: vinorelbine 25 mg/m^2^ day 1 and 8; cisplatin 75 mg/m^2^ day 1 repeated every 3 weeks until the end of RT. A total of three cycles was delivered. Following this therapy he was in good health, his regular follow-up evaluations did not reveal any notable pathologic findings, and he did not need to take any specific medication. However, 10 years later he presented a right scapula pain. He stopped smoking tobacco in 2007, and suffered from right scapula pain for 3 months. On admission his temperature was 37.5, his pulse 90 beats per minute, and his blood pressure 135/75 mmHg. A clinical examination found a fixed, sensitive right scapular mass that measured 10 cm at its big axis (Fig. 2). A neurologic examination showed a conscious patient well oriented with preserved memory. A cranial nerve examination showed no disturbance in olfaction or in visual fields. Ocular motricity is well conserved as well as facial sensation and motricity. The rest of the cranial nerve examination did not reveal any dysarthria, hearing loss, tongue weakness, or neck weakness. Motricity of arms and inferior members was well conserved without any involuntary movements. Muscular force was preserved as well as sensitivity (light touch, temperature, vibration, joint position sense). He suffered from a slight neuropathic pain of his right arm whose intensity was estimated to be 4/10 according to the visual analogue scale. Biceps, triceps, brachioradialis, knee (patellar), and ankle (Achilles) reflexes did not reveal any abnormalities. A chest computed tomography (CT) scan showed a tumor process on his right scapula, with sequela lung injury in the territory of the first tumor (Fig. 3). Magnetic resonance imaging (MRI) of the scapular region showed malignant tumor process centered on the supraspinous and infraspinous muscles with hyposignal at T1 and heterogeneous hypersignal at T2 measuring 90 mm on the longest axis and 60 mm on the shortest axis. There was no involvement of his right shoulder joint (Fig. 4). Data from a bone scintigraphy were in favor of a hyperfixation focus at the right scapula (Fig. 5). A single-photon emission computed tomography (SPECT)-CT examination showed an osteocondensing and lytic aspect in the spine of his right scapula (Fig. 6). Laboratory tests did not reveal any abnormalities, showing good liver and renal functions. A surgical biopsy of the tumor was performed. Histopathological examination showed a grade 1 chondrosarcoma (Fig. 7). An extension assessment, including CT of his chest and abdominal ultrasonography, did not find distant metastasis. After a multidisciplinary staff meeting a surgical resection was performed with safe margins. The surgical procedure consisted of a resection of his right scapula, with fixation of his right arm thanks to osteosynthesis equipment. No adjuvant treatment was necessary because of the free margins after surgery and the low grade of the chondrosarcoma. A follow-up at 6 months (clinical examination and CT scan) did not show any sign of recurrence or specific complication.

## Discussion

This is a rare case of radiation-induced chondrosarcoma, occurring 10 years after chemoradiation for non-small cell lung cancer (NSCLC). RIS after chemoradiation for lung cancer is a very rare complication. The rarity of this situation is due to the poor prognosis of advanced stage of NSCLC with limited survival, which exceptionally exceeds 3 years [1].

The carcinogenic effects of ionizing radiation have been described in several publications [5]. The real mechanisms in radiation-induced tumor genesis remain poorly known [6]. In a published series, their frequency varies between 0.15 and 0.75% [5, 6]. Phillips and Sheline estimated the frequency of sarcomas after irradiation for breast cancer to be 0.23% [6]. Mark *et al.* estimated the absolute risk of developing RIS to be from 0.03 to 0.8% after RT for gynecologic malignancies [7]. Amendola *et al.* noted an estimated incidence of sarcomas of 0.09 to 0.11% after radiotherapy for any purpose [6]. Furthermore, Huvos *et al.* and Souba *et al.* estimated that 5% of sarcomas developed after therapeutic or accidental irradiation [4]. All tissue types can be processed by irradiation, although the radio sensitivity varies with the type of irradiated organ [8]. There is no histological evidence to confirm the origin of radiation-induced tumors [9]. Ionizing radiation causes damage to healthy tissue included in the radiation field. Some authors identified the specific mesenchymal stem cells that can regenerate on injury [7, 9]. On irradiation-induced injury, the stem cells rapidly start proliferating and can regenerate injured tumoral tissue [7].

Several risk factors are probably implicated in influencing the occurrence of these secondary tumors. The radiation dose which is a variable parameter depending on the irradiated organs, the type of chemotherapy that can potentiate the effect of RT, young age (children are especially sensitive to radiation oncogenesis), and a genetic predisposition to multiple tumors [9, 10]. Young age exposure seems to be an important risk factor, especially in tumors with a good prognosis such as hematologic malignancies [10]. Relative risk (RR) of second solid cancer at high-dose sites for radiotherapy in lung cancer is different depending on the period of latency. For latency between 5 and 9 years the RR is estimated to be 1.12 (0.98–1.27), between 10 and 14 years it is estimated to be 1.37 (1.12–1.65), and after 15 years of latency the RR is estimated to be 1.62 (1.23–2.09) with p-trend at 0.0079 [11]. There are no clear data reporting eventual implication of concurrent chemotherapy in the carcinogenesis of RIS.

### Diagnosis

The diagnosis is established by criteria established by Cahan *et al.* in 1948 [12] and revised by Murray *et al.* in 1999 [13]. A history of radiotherapy for cancer, an asymptomatic latency period of several years, the occurrence of tumor in the irradiation field, and histological evidence of the secondary tumor [12]. Complete remission of the primary tumor is also necessary to establish this diagnosis [9]. Our case meets the criteria of Murray *et al.* for RIS [13]. They specify that radiotherapy must have been administered previously and that the sarcoma must have developed from an area within the 5% isodose line [14]. To the best of our knowledge, radiation-induced scapular chondrosarcoma has not been described previously. The rarity of radiation-induced chondrosarcoma is reflected by the very few case series that have included such cases. In fact, in a multi-institutional series of 80 histologically confirmed cases that were diagnosed as RIS between 1975 and 1995, only one patient had chondrosarcoma [14]. Some reviews reported a large predominance of osteosarcomas and fibrosarcomas in RIS of the chest wall [11].

### Management

Therapeutic approaches are often limited. Surgery is the only curative method, when it is possible. The difficulty of surgery in irradiated areas is known, but must be seriously considered if the tumor is diagnosed at an early stage and if the surgeon is experienced. In advanced stages, chemotherapy may have a place, despite poor tumor response in irradiated areas [15]. In these cases the prognosis is also poor, and median survival does not exceed a few months. The use of new irradiation procedures reducing the field of exposure should allow a clear decrease in the incidence of RIS [15].

## Conclusions

This case is about a very rare situation, radiation-induced chondrosarcoma of the right scapula. This event is very uncommon making it almost impossible to perform prospective clinical trials specifically designed to compare different treatment approaches. Surgery is the principal method of treatment. A close follow-up of irradiated patients is the only way for an early diagnosis of this serious complication.
